# Use of a molecular bacterial load assay to distinguish between active TB and post-TB lung disease

**DOI:** 10.5588/ijtld.21.0459

**Published:** 2022-03-01

**Authors:** P. M. Mbelele, W. Sabiiti, S. K. Heysell, E. Sauli, E. A. Mpolya, S. Mfinanga, S. H. Gillespie, K. K. Addo, G. Kibiki, D. J. Sloan, S. G. Mpagama

**Affiliations:** 1 Kibong’oto Infectious Diseases Hospital, Sanya Juu, Siha, Kilimanjaro, Tanzania; 2 Department of Global Health and Biomedical Sciences, School of Life Sciences and Bioengineering, Nelson Mandela African Institution of Science and Technology (NM-AIST), Arusha, Tanzania; 3 Division of Infection and Global Health, School of Medicine, University of St Andrews, St Andrews, Scotland, UK; 4 Division of Infectious Diseases and International Health, University of Virginia, Charlottesville, VA, USA; 5 National Institute for Medical Research (NIMR), Muhimbili Center, Dar es salaam, Tanzania; 6 Department of Bacteriology, Noguchi Memorial Institute for Medical Research, University of Ghana, Accra, Ghana; 7 East African Health Research Commission (EAHRC), Bujumbura, Burundi

Dear Editor,

We report new diagnostic findings showing that a TB molecular bacterial load assay (TB-MBLA) can distinguish between patients with active TB and post-TB lung disease (PTLD). PTLD is defined as evidence of respiratory disability without active TB among patients who clinically present with chronic respiratory symptoms (e.g., cough and difficulty in breathing) after previous bacteriologically confirmed TB treatment.^1^,^2^ Several reports from various settings have described patients with PTLD returning a positive test result for active infection using Xpert^®^ MTB/RIF (Cepheid, Sunnyvale, CA, USA) testing.^2^–^5^ For example, Ngabonziza et al. reported false-positive Xpert results in 34% of patients in Rwanda,^4^ whereas Sekyere et al. and Yang et al. reported respectively 45% in South Africa and 46% in China.^6^,^7^ The Xpert assay simultaneously detects the presence of DNA of *Mycobacterium tuberculosis* complex (MTBC) and rifampicin-associated mutations in the *rpo*B gene. Since its endorsement in 2010 by the WHO, the time from diagnosis to treatment of rifampicin-resistant/multidrug-resistant TB (RR/MDR-TB) in Tanzania has decreased from 9 months to less than 2 months.^8^ However, in individuals being investigated for recurrent TB, Xpert is unable to differentiate between the DNA of live or dead MTBC, leading to false-positive results.^9^ The DNA of MTBC is stable and may be detected using Xpert up to 5 years after TB treatment.^9^ Previous attempts to distinguish between live and dead DNA of MTBC using Xpert included pre-treating sputum samples with propidium monoazide (Biotium Inc, Hayward, CA, USA), a chemical that selectively intercalates dead DNA and therefore inhibits its amplification and detection.^10^ This approach had low specificity, at 71%, for detecting active TB compared to the gold standard mycobacterial culture.^10^ From a clinical point of view, it has been suggested that individuals with low or very low MTBC false-positive Xpert results require a repeat test using another specimen.^4^,^11^ Other groups have withheld anti-TB medications and trialed broad-spectrum antibiotics to determine symptomatic response before further TB investigation.^12^

TB-MBLA is a quantitative reverse transcription PCR (RT-qPCR) test that uses 16S rRNA to detect and quantify viable MTBC strains. Previous longitudinal studies among patients treated for RR/MDR and drug-susceptible TB showed an overall moderate positive correlation in time to sputum conversion from positive to negative between TB-MBLA and solid Löwenstein-Jensen (LJ) culture (*r* = 0.46, 95% CI 0.36–0.55); *P* < 0.001)].^13^ Similarly, another study demonstrated an increase in correlation between TB-MBLA and MGIT liquid culture in detecting active TB from 0.66 (95% CI 0.56–0.74) pre-treatment to 0.73 (95% CI 0.44–0.89) at Month 1 of treatment.^14^

The need to develop an optimal approach for discriminating between PTLD and recurrent TB has recently been prioritised.^15^ To address this, we integrated TB-MBLA into a routine TB testing algorithm. In 2019, we consecutively recruited 41 adult patients aged ≥18 years, including 8 patients with RR-susceptible and 33 patients RR/MDR-TB who were referred to Kibong’oto Infectious Diseases Hospital from various clinics with positive MTBC detected using Xpert. The quantity of MTBC was scored as high, medium, low and very low if detected using Xpert at a cycle threshold (CT) value of respectively <16, 16–22, 23–28 or >28. Inclusion criteria were evidence of chronic respiratory symptoms, including cough, chest pain with or without haemoptysis and weight loss with a positive Xpert result 1 year after completing TB treatment. Prior to study procedures, all patients provided informed consent approved by the National Institute for Medical Research in Tanzania (NIMR/HQ/R.8a/Vol. IX/2662). Patients provided one pre-treatment early morning sputum sample for testing using smear microscopy (SM), LJ culture, GenoType MTBDR*plus* (Hain Lifesciences, Nehren, Germany) and TB-MBLA, as previously described.^5^,^13^ We also performed GenoType Mycobacterium CM v2.0 (Hain Lifesciences) testing for non-tuberculous mycobacteria (NTM) detection in samples with positive LJ-culture result but negative on MTBDR*plus* and TB-MBLA. Based on original Xpert results, all patients received first- or second-line anti-TB treatment regimens in accordance with Tanzania guidelines.

The sociodemographic and clinical characteristics, and microbiological results of 41 previously treated individuals are shown in the Table. Among 41 patients, 23 had high or medium MTBC result. Of these, respectively 16 (70%), 20 (87%) and 23 (100%) had TB by positive SM, LJ-culture and both MTBDR*plus* and TB-MBLA. The remaining 18 (44%) patients had very low MTBC quantity from the original Xpert result. This very low MTBC burden was common in patients with PTLD compared to those with active TB disease (15, 100% vs. 3, 12%; *P* < 0.001). Active TB in three patients with very low MTBC burden on the original Xpert result was confirmed using both LJ culture and TB-MBLA (*n* = 1) and TB-MBLA (*n* = 2), and all had cavitary disease on chest radiograph (Table). Low bacterial burden could partially be attributed to immunodeficient-disease severity in two malnourished patients (body mass index: 14.9 kg/m^2^ and 16.3 kg/m^2^) and in a person living with HIV/AIDS (*n* = 1).^16^ Overall, 15/41 (36.6%) patients in our cohort had no TB detectable by any other tests, and were therefore presumed to have PTLD. TB-MBLA results were 93% (95% CI 82–99) concordant with LJ-culture in discerning active TB and non-TB disease in 41 patients. There were discordant results from three patients with low MTBC burden, including two patients who had positive TB-MBLA, but negative LJ Culture. It is evident that this discordance can be attributable to 1) the presence of viable MTB detectable using TB-MBLA, but not culturable; 2) the use of solid LJ, which is less sensitive than liquid culture; 3) sampling error; and 4) unclearly established survival time of RNA in sputum after TB treatment. One patient had positive culture but negative TB-MBLA, and was later confirmed to have *M. intracellulare*, a common NTM species in previously treated patients, which may contribute to PTLD pathology. Moreover, 8/15 (53%) patients had prior exposure to silica dust, with chest radiological features suggestive of silicosis. A combination of previous TB treatment and silicosis could also explain the reported chronic respiratory symptoms in patients with PTLD.

**Table:** Clinical characteristics, microbiological results and treatment outcomes of 41 previously treated individuals

Baseline characteristic		Overall (*n* = 41) *n* (%)	TB (*n* = 26) *n* (%)	Presumed PTLD (*n* = 15) *n* (%)	*P* value
Age, years, mean ± SD		41 ± 12	38 ± 11	46 ± 12	0.044
Sex	Male	31 (76)	19 (76)	12 (76)	0.719
	Female	10 (24)	7 (24)	3 (24)	
HIV status	Positive	12 (29)	9 (31)	3 (20)	0.479
	Negative	29 (71)	17 (69)	12 (80)	
Silicosis	Yes	18 (44)	7 (27)	11 (73)	0.022
	No	23 (56)	19 (73)	4 (27)	
Cigarette smoking	Yes	21 (51)	13 (50)	8 (53)	0.993
	No	20 (49)	13 (50)	7 (47)	
Number of TB episodes prior to current diagnosis	1	24 (59)	16 (61)	8 (53)	0.898
	2	12 (29)	7 (27)	5 (34)	
	≥3	5 (12)	3 (12)	2 (13)	
Time since the last TB treatment, years, median (min - max)		3 (1–19)	3 (1–17)	2.5 (1–19)	0.693
Chest radiological features	Normal	1 (2	0 (0)	1 (7)	<0.001
	Cavities	17 (42)	17 (65)	0 (0)	
	Cavity and fibrosis	10 (24)	8 (31)	2 (13)	
	Fibrosis	13 (32)	1 (4)	12 (80)	
Smear microscopy results	Positive	18 (44)	17 (65)	1 (7)	<0.001
	Negative	23 (56)	9 (35)	14 (93)	
LJ culture results	Positive	24 (59)	23 (88)	1 (7)	<0.001
	Negative	17 (41)	3 (12)	14 (93)	
Xpert score	High	13 (32)	13 (50)	0 (0)	<0.001
	Medium	9 (22)	9 (35)	0 (0)	
	Low	1 (2)	1 (4)	0 (0)	
	Very low	18 (44)	3 (12)	15 (100)	
Line-probe assay	Positive	23 (56)	23 (88)	0 (0)	<0.001
	Negative	18 (44)	3 (12)	15 (100)	
TB-MBLA	Positive	23 (56)	23 (88)	0 (0)	<0.001
	Negative	18 (44)	3 (12)	15 (100)	
Programmatic treatment outcomes	Cured	29 (71)	18 (69)	11 (73)	0.138
	Completed	10 (24)	8 (31)	2 (13)	
	Failed	1 (2)	0 (0)	1 (7)	
	Died	1 (2)	0 (0)	1 (7)	

PTLD = post-TB lung disease; SD = standard deviation; LJ = Löwenstein-Jensen; MBLA = molecular bacterial load assay.

Our results support the use of TB-MBLA to distinguish between active TB and non-TB disease, including PTLD, among symptomatic patients being investigated for recurrent TB and presenting with a low/very low Xpert test results. PTLD is an important medical condition that is under-recognised in high TB burden settings such as Tanzania. Our prior survey to determine the burden of PTLD revealed chronic respiratory symptoms and bronchitis in respectively 98 (45%) and 24 (11%) of 219 people with prior TB treatment.^1^ These findings support the integration of TB-MBLA in diagnostic tests to distinguish between TB and PTLD in individuals with low MTBC burden and chest fibrotic changes. This may be of even further value when using the Xpert Ultra (Cepheid) platform, with its higher sensitivity but limitations in identifying “trace” results as true-positives.^3^ Given the small sample size tested here, validation of a proposed TB-MBLA algorithm would require a prospective study that includes evaluating its accuracy and clinical outcomes.
